# Enhanced Activation of Persulfate by Co-Doped Bismuth Ferrite Nanocomposites for Degradation of Levofloxacin Under Visible Light Irradiation

**DOI:** 10.3390/ma12233952

**Published:** 2019-11-28

**Authors:** Xin Zhong, Zheng-Shuo Zou, Hu-Lin Wang, Wei Huang, Bin-Xue Zhou

**Affiliations:** Department of Environmental Science and Engineering, Beijing Normal University, Zhuhai 519000, China; zzsslq22@163.com (Z.-S.Z.); wanghulin.hu@163.com (H.-L.W.); hw_david@21cn.com (W.H.);

**Keywords:** bismuth ferrite, levofloxacin, persulfate, visible light

## Abstract

In this study, magnetic visible light driven photocatalysts (bismuth ferrite, Bi_2_Fe_4_O_9_, BFO and Co-doped bismuth ferrite, Co-BFO) were successfully prepared by the facile hydrothermal method. The catalyst was used in the application of heterogeneous persulfate (PS) system under visible LED light irradiation for the degradation of levofloxacin (LFX), proving to be an excellent photocatalyst when evaluated by various characterization methods. The effect of Co-doping in the BFO structure was investigated that the decrease of band gap width and the generated photoelectrons and holes would effectively reduce the recombination of photogenerated electron-hole pairs, leading to the enhancement photocatalytic activity. The results demonstrated that Co-BFO catalyst had a high photodegradation efficiency over a wide pH range of 3.0–9.0 and the Co-BFO-2 composite displayed the optimal catalytic performance. It was found that the degradation rate of LFX by Co-BFO-2 catalyst was 3.52 times higher than that of pure BFO catalyst under visible light condition. The free radical trapping experiments and EPR tests demonstrated that superoxide, photogenerated holes and sulfate radicals were the main active species in the photocatalytic degradation of LFX. And a possible photocatalytic degradation mechanism of LFX was proposed in the Vis/Co-BFO/PS process. These findings provided new insight of the mechanism of heterogeneous activation of persulfate by Co-BFO under visible light irradiation.

## 1. Introduction

In recent years, the water pollution problems are more severe, which have received more concerns by many researchers, due to the various containments such as dyes, pesticides, pharmaceuticals and herbicides [1,2,3]. Those pollutants are usually toxic, stable and persistent in nature, which showed almost no biodegradability and hard to be eliminated by conventional treatment technologies [4,5,6]. Advanced oxidation processes (AOPs) have attracted much more attention due to its remarkable removing efficiency of these contaminants which are highly desired for its moderate and easy operation reaction [7,8,9]. Among various AOPs, heterogeneous photocatalytic processes have been regarded as one of the most advantageous technologies for the removal of containments, due to its high efficiency, operation simplicity and reusability [10,11,12]. The photocatalysts played unique role in the photocatalysis process, which have been studied by researchers, such as CuO [13], Fe_2_O_3_ [14], g-C_3_N_4_ [15] and BiOCl [16]. The investigation of visible-light-driven photocatalysts was of great importance for the utilization of visible light. 

Among these photocatalysts, Bi_2_Fe_4_O_9_ (BFO) was considered as one of potential visible-light-driven materials with ferroelectric properties and narrow band gap [17,18,19,20]. The utilization of BFO in heterogeneous photocatalysis was studied by many researchers. Unfortunately, the photocatalytic reaction rate and efficiency of reported BFO was not satisfied due to the quick recombination of photogenerated electrons and holes. Herein, various works have been reported that metal doping could be an effective strategy to improve the photocatalytic efficiency by the substitution of iron element in pure BFO, leading to the extended response in visible light region [21,22,23]. Inspired by these works, Co species doping in the structure of BFO not only regarded as reactive sites for the photocatalysis process, but also facilitated the improvement of optical properties via electron transfer. It was assumed that cobalt (Co) element could be one promising element that doped into BFO as reported by other researches [24]. As cobalt element doping materials showed excellent electrochemical properties, the light absorption region of BFO could be extended and expected high photocatalytic activity. 

However, most of the photocatalytic oxidation reaction took extensively long reaction time to reach the satisfied contaminate removal efficiency. Hence, the photocatalytic process could combine with other AOPs or the addition of oxidants species to enhance the activity and reduce the recombination of charge carrier. Sulfate radical-based AOPs have obtained more and more attention as one effective technology for the degradation of pollutants, which has higher redox potential, longer half life time and no selectivity in a wide pH value in comparison with hydroxyl radicals [25,26,27]. Some earlier works have reported the introduction of persulfate (PS)/peroxymonosulfate (PMS) to the photocatalysis by using bismuth ferrite as photocatalyst (Table S3). To our best knowledge, limited research has been reported on the combination with PS and Co-BFO under visible light for the treatment of antibiotics wastewater, especially using light-emitting diode (LED) as the light source. 

Herein, we have synthesized pure BFO and Co doped BFO photocatalyst using the one-pot hydrothermal method. The effect of Co doping in BFO are systematically investigated by XRD, SEM, TEM, XPS, VSM and UV-vis characterization methods. In recent years, the threats of antibiotics drugs have obtained arising attention which the residual concentration in the ground water would cause the increment risk of emergence and spread of resistant bacteria. Levofloxacin (LFX) was one kind of broad-spectrum antibiotics which possessed limited degradability in the conventional biological removal processes, leading to negative effects on microorganism communities and human beings. So, there were of great importance to deal with LFX-containing water to minimize the risk. LFX was chosen as the target pollutant in the heterogenous PS system under visible light irradiation by using the Co-BFO as catalyst. The active species were detected and a possible LFX degradation mechanism was given. Moreover, the effect of experimental factors on the photocatalytic degradation of LFX was investigated.

## 2. Materials and Methods

### 2.1. Synthesis of BFO and Co-BFO Catalysts

All the chemicals and reagents were of analytic grade and used without further purification. The BFO and series different Co doping samples of Co-BFO were prepared by one-pot hydrothermal method. Firstly, 4.85 g Bi(NO_3_)_3_·5H_2_O was extensively dissolved in 3M HNO_3_ solution. Then, different amount of Fe(NO_3_)_3_·9H_2_O and Co(NO_3_)_2_·6H_2_O was added into the solution. 8 M NaOH was dropped slowly into the obtained solutions under vigorous stirring. The precipitates were transferred to an autoclave at 140 °C for 6 h. Finally, the samples were centrifuged and washed, dried at 60 °C for 24 h. In the BFO preparation, the amount of Fe(NO_3_)_3_·9H_2_O was 4.04 g according to the chemical ratio. For comparison, series samples of Co-BFO were also synthesized by the same method through changing the molar ratio of Fe/Co, which denoted as Co-BFO-1 (58.26 mg Co amount), Co-BFO-2 (116.52 mg Co amount), Co-BFO-3 (174.78 mg Co amount) and Co-BFO-4 (291.3 mg Co amount), which was shown in Table S2. Pure BFO sample was also synthesized without the cobalt addition.

### 2.2. Characterization of Catalysts

Powder X-ray diffraction (XRD) pattern analysis was obtained using a Smart Lab 9 kW (Rigaku, Tokyo, Japan) diffractometer coupled with a graphite monochromatic Cu Kα radiation (λ = 1.54 Å) at the accelerating voltage 40 kV and the current 30 mA over the 2θ scanning range of 10–80°. Photoelectron spectroscopy (XPS) analysis was performed using an X-ray photoelectron spectroscopy (Thermo Fisher ESCALAB250Xi, Karlsruhe, Germany) spectrometer with a monochromatized Al-K X-ray source. The UV-vis spectra were performed on Lambda950 (PerkinElmer, Norwalk, Connecticut, USA) Plus instrument. The morphology and chemical composition of the catalyst were characterized by a SIGMA 500/VP (ZEISS, Oberkochen, Germany) scanning electron microscope (SEM). The high-resolution transmission electron microscopy (HR-TEM) images were obtained on a JEM-2100F (Tokyo, Japan) transmission electron microscopy. And the surface elementary compositions of Co-BFOs were achieved by energy dispersive spectrometer mapping (EDS) and ICP-MS (Agilent 7000, Palo Alto, California, USA). N_2_ adsorption/desorption analysis and pore size distribution were measured on an ASAP 2460 instrument (Micromeritics, Atlanta, Georgia, USA). Magnetic curve measurements were performed with SQUID-VSM instrument at room temperature.

### 2.3. Experimental Procedure

For the LFX removal experiments, all the experiments were conducted in a 100 mL cylindrical reactor with 50 mL LFX solution (15 mg L^−1^) at room temperature which was showed in our previous study. The LED lamp with the wavelength close to 450–460 nm was used in all experiments, and the light intensity could be regulated to be 50 mW/cm^3^. The gap distance between the reactor and LED light was around 3 cm. The initial pH was adjusted by 0.1M H_2_SO_4_ or 0.1M NaOH. Before the LED light turned on, 0.025 g catalyst was suspended into the LFX solution for at least 30 min stirring in order to reach the absorption equilibrium. As soon as the desired persulfate (DaMao Company, Tianjin, China) was added in the solution, the LED lamp was turned on with a constant stirring speed of 500 rpm. At the predetermined time interval, 1 mL solution samples were regularly selected and filtered through 0.22 μm PTFE syringe filter which were quenched with methanol before analyzing the residual LFX concentration. Three runs for each experiment were repeated, and mean values were used.

### 2.4. Analytical Methods 

Total organic carbon (TOC) analyzer (Elementar vario) was used to analyze the mineralization of LFX. Inductively coupled plasma-Mass Spectrometry (ICP-MS, Agilent 7000, Palo Alto, California, USA) was used to determine the Fe and Co leaching concentration. The concentration of LFX was monitored by HPLC (Agilent 1100 LC/MSD, C8 column, 293 nm, Palo Alto, California, USA). Electron spin paramagnetic resonance (EPR) spectra was achieved to detect radical species by using 5, 5-dimethyl-1-pyrroline-N-oxide (DMPO) as the radical-trapping reagent on a Bruker EMXPlus-10/12 instrument in deionized water. In the batch experiments, the degradation of LFX was fitted well by the pseudo first order kinetics, which can be express as Equation (1).

ln C/C_0_= −k_obs_ × t, (1)

where C refer to the concentration of LFX at time t, C_0_ refer to the initial LFX concentration, k_obs_ refer to the kinetic rate constant, and t refer to the reaction time.

## 3. Results

### 3.1. Catalyst Characterization 

**Figure 1:**
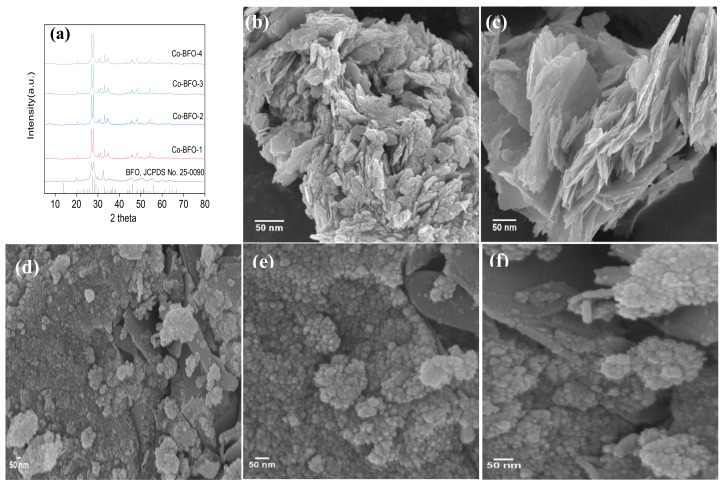
(**a**) X-ray diffraction (XRD) patterns of BFO and Co-BFO samples; (**b**,**c**) SEM images of BFO and (**d**–**f**) Co-BFO-2.

The XRD spectra of the pure BFO and Co-BFO were shown in Figure 1a. The XRD pattern of pure BFO is in well agreement with the structure of BFO (JCPDS Card No. 25-0090), indicating that the single phase of BFO was successfully synthesized. In the case of Co-BFO samples, it was observed that no apparent peaks of iron oxides and cobalt oxides or other phases could be detected, suggesting that the doping Co hardly affect the host structure of BFO. The XRD peaks for Co-BFO samples were slightly shifted to right side angle, due to the higher radius of Fe (0.0645 nm) than that of Co (0.0545 nm) and the shrinkage of unit volume [28]. This implied that Co element could take place of Fe and incorporated into the host structure, which could be evidenced by the estimation of unit cell parameters of these samples as shown in Table S1. It was found that the unit cell volumes decreased with increasing concentration of Co element. It was reasonable to resume the shrinkage of the unit cell volume when Fe was substituted by Co element, indicating the Co element successfully doped into BFO lattice. The Co-BFO catalyst were successfully synthesized by one-pot hydrothermal method which showed almost no weaken crystallinity of pure BFO. The Rietveld refinement of BFO and Co-BFO-2 was obtained and showed in Figure S1. The refinement date indicated the BFO and Co doped BFO were in well agree with the real structure. After the Co doping, the refined data indicated that 1.39% of Fe was substituted by Co, which was close to the doping ratio obtained by ICP.

Figure 1b–f shows the SEM images of the as-synthesized BFO and Co-BFO-2 samples. The SEM of pure BFO (Figure 1b,c) appeared almost nanoplate-shaped with the particle diameter of 40–50 nm, which was in accordance with the XRD data. In addition, the Co-BFO-2 particles exhibited fine microspheres from the agglomeration ranging from 20–40 nm. By comparison, after Co element doping, the particle size of the catalysts became slightly smaller than the undoped BFO, which might attribute to the increment of nucleation rate and inhibition of crystallization.

**Figure 2:**
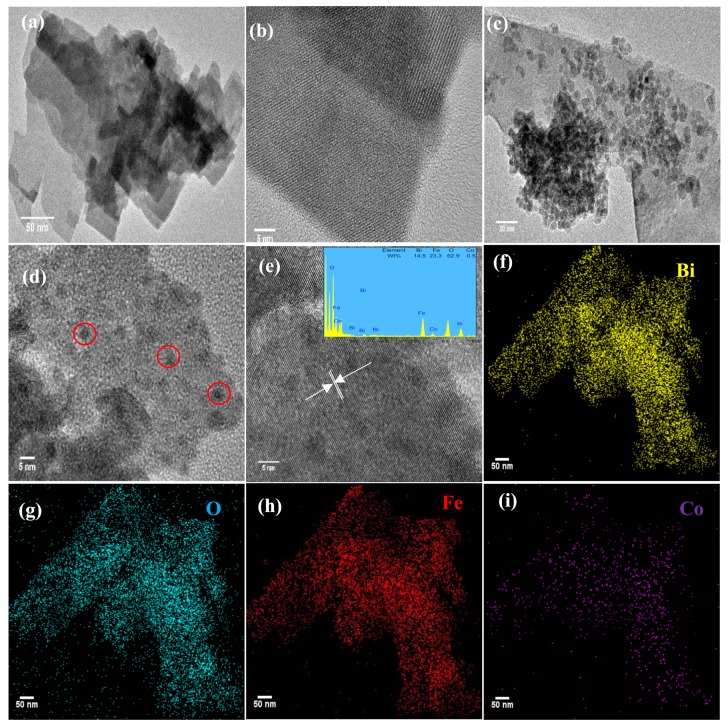
TEM images of BFO (**a**,**b**); Co-BFO-2 (**c**–**e**) and EDS mapping of Co-BFO-2 (**f**–**i**).

Moreover, in order to investigate the specific characteristics about the morphology of the catalyst, TEM and EDS mapping observation was employed and shown in Figure 2. It was observed that the Co-BFO-2 exhibited smaller particle size than that of BFO. In addition, both of BFO and Co-BFO-2 catalyst showed a slight agglomeration phenomenon. It was be seen that the lattice fringes with d-spacing of 0.547 nm corresponding to (110) facets of BFO, illustrating the high crystallization of the product. In order to further determine the composition of Co-BFO-2, EDS element mapping was recorded which observed that all the elements were presented in the synthesized catalysts and well distributed in the zone. The atomic ratio of Fe and Co was 23.3:0.5 in Co-BFO-2 samples, which agreed with the theoretical chemical composition, demonstrating the presence of Co elements homogeneous exists in the products.

**Figure 3:**
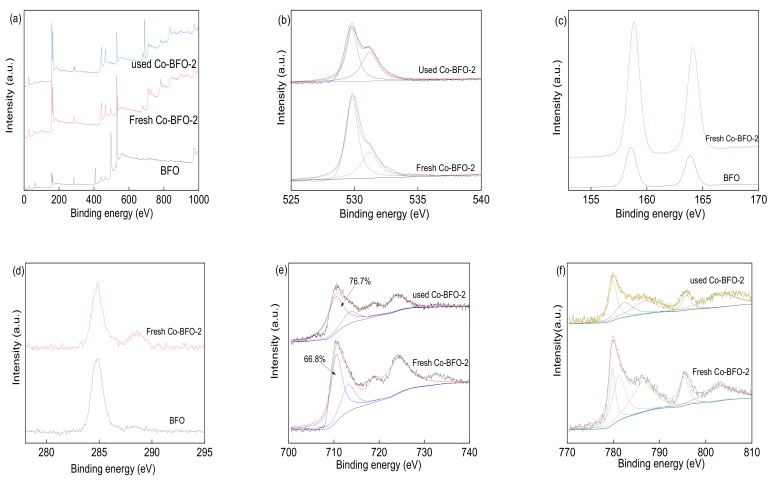
X-ray photoelectron spectroscopy of BFO and Co doped BFO (Co-BFO-2): survey spectra (**a**), O 1s (**b**), Bi 4f (**c**), the standard C 1s (**d**), Fe 2p (**e**), Co 2p (**f**) level spectra.

The composition and chemical status of BFO and Co-BFO were conducted by XPS to make a better insight into the role of element species, which shown in Figure 3. The full scale XPS spectrum of BFO and Co-BFO clearly showed the peaks of Bi, Fe, O and Co elements, and the binding energy of each element was amended by C 1s peaks to 284.6 eV. Figure 3c exhibited the XPS spectra of Bi 4f region with peaks of 158.6 eV and 163.9 eV, the splitting energy was 5.3 eV which could be addressed to Bi 4f_7/2_ and Bi 4f_5/2_, respectively. It can be seen that the Bi 4f peaks of Co-BFO-2 catalyst shifted to higher binding energy (~0.2 eV) than that of BFO, which might belong to the reduced distance of Bi and O element by Co doping modification [29]. In the O 1 s XPS spectra (Figure 3b), the peaks could be fitted into two bands of 529.8 and 531.2 eV, which could be assigned to the lattice oxygen in the catalyst and absorbed water on the surface. After the photocatalytic reaction, the peak ratio of 531.2 eV in the used Co-BFO-2 to the peak of 529.8 increased from 29.6% to 44.9%, which would be assigned to absorbed hydroxyl groups on the surface. In addition, the content of absorbed water decreased from 38% to 33%, which verified the hydroxyl groups generated from the absorbed H_2_O molecule in the PS activation process under visible LED light irradiation. The Fe 2p XPS spectra (Figure 3e) obviously observed three main peaks at 710.0, 712.2 and 717.3 eV, which would be assigned to Fe(II) and Fe(III).

As shown in Figure 3f, the Co 2p spectra split to Co 2p_1/2_ (795.3 eV) and Co 2p_2/3_ (781.3 eV) peak followed by shakeup satellites (786.1 eV), suggesting the existing of the Co(II) and Co(III). For the used Co-BFO-2, the characteristic peaks of Co 2p were not changed before and after catalytic PS activation under visible LED light irradiation. It was reasonable to expect the participation of Co species in the photocatalyst reaction. It was observed that the binding energies of used Co-BFO-2 shifted slightly to the higher binding energies compared to that of pristine Co-BFO-2, implying the possible existence and participation of Fe and Co in PS activation. The results indicated that the Co-BFO-2 catalyst was very stable and recyclable.

**Figure 4:**
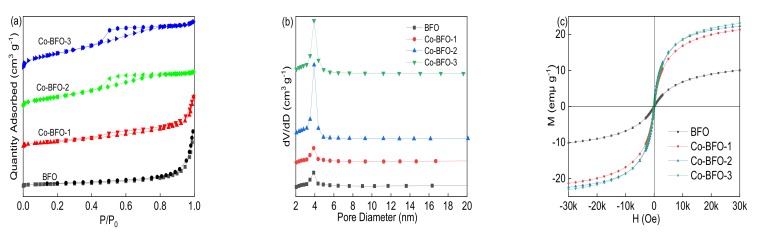
(**a**) N_2_ adsorption-desorption isotherm, (**b**) pore size distribution and (**c**) magnetic hysteresis loops at room temperature for BFO and Co-BFO samples.

Figure 4a,b showed the N_2_ absorption/desorption isotherm and pore size distribution of BFO and Co-BFO samples. The BET surface area of BFO was 11.8 m^2^ g^−1^ and the pore size was around 3.8 nm, while the BET surface area of Co-BFO-2 was 58.17 m^2^ g^−1^ and pore size was 4.24 nm, which was applied in the further photocatalysis reaction. In addition, with more addition of Co element content, the BET surface area increased and the pore size became smaller than that in the pure BFO catalyst, which listed in Table 1. The isotherms of both BFO and Co-BFO samples showed type H3 isotherms [30]. This observation suggested that the BET surface area was significantly improved by the medication with Co species, which was expected higher adsorption capacity than that of pure BFO. The magnetic property of BFO and Co-BFO samples was examined by VSM at room temperature in the range of −3.0 T to 3.0 T. As can be seen in Figure 4c, the M-H curve of BFO and Co-BFO-2, the saturation magnetization value was 9.76 emμ g^−1^ and 22.17 emμ g^−1^. Moreover, the saturation magnetization value was increased by the addition of Co element (Table 1), which was enough for magnetic separation by magnet. It was observed that the Co-BFO samples exhibited well magnetic property than that of pure BFO.

**Figure 5:**
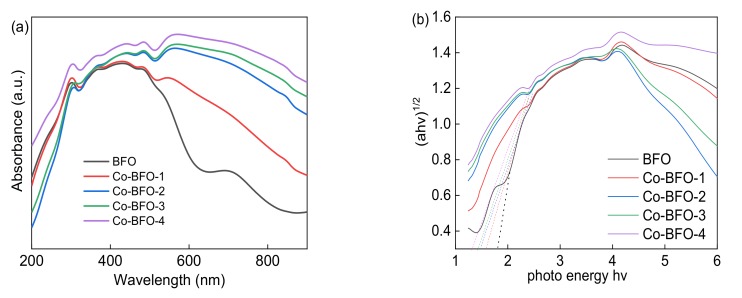
(**a**) UV–visible absorption spectra and (**b**) band gaps of BFO and Co-BFO samples.

As reported by researchers, the BFO possessed narrow band gap around 2.0 eV, which might perform under the visible absorption range. As can be seen from Figure 5, Co-BFO materials showed a long absorption tail in the visible-light range. The absorption edge was observed a red shift compared to the pure BFO. On the basis of UV-vis spectrum, the band gap of a semiconductor can be calculated using the following equation [31,32,33].

αhυ= A (hυ −Eg) ^n/2^, (2)

where α, hv, A and Eg, are the absorption coefficient, photon energy, a constant and the band gap, respectively. The band gap energy was calculated from the Tauc plot of (αhv)^0.5^ versus photon energy (hv), which was calculated as 1.83, 1.63, 1.50, 1.44 and 1.34 eV, respectively. As the amount of Co element increased, the band gap value decreased and the color of samples grew deeper, resulting in better photocatalytic performance under visible light irradiation.

### 3.2. Photocatalytic Procedures

**Figure 6:**
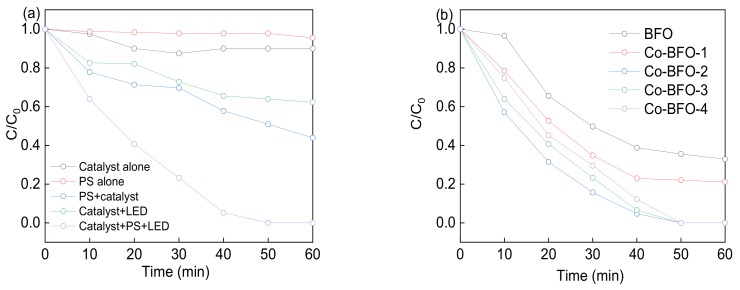
(**a**) Degradation of LFX under visible light irradiation in different systems, (**b**) different Co loading effect on the LFX degradation. Reaction conditions: [LFX] = 15 mg L^−1^, [Co-BFO] = 0.5 g L^−1^, [PS] = 0.2 mM.

The catalyst activities were firstly evaluated by the degradation of LFX in the heterogenous PS activation process under visible light. As shown in Figure 6a, more than 99% of LFX was degraded in 60 min by Co-BFO-2/PS under visible light irradiation. Adsorption of LFX on the catalyst and direct oxidation of LFX by PS were excluded before the degradation experiments as the removal efficiency was less than 10% in 60 min. Direct photolysis of LFX by visible LED light were almost negligible due to the limited absorption activity of LFX under visible light with the wavelength around 293 nm. Approximately 37.7% of LFX were oxidized in 60 min by photocatalysis system. It was reported that LFX was degraded by the presence of hydroxyl radicals and sulfate radicals by the splitting of PS and photogenerated holes and superoxide from Co-BFO as the photocatalyst. When the catalyst and PS both presented in the solution which could be regarded as heterogenous PS process, the LFX degradation efficiency was slightly improved, and 56.1% of LFX was oxidized after 60 min reaction. Moreover, a synergistic action between Co-BFO and PS under visible light was observed. LFX degraded quickly in the presence of sulfate radicals, hydroxyl radicals, photogenerated holes and superoxide, resulting in complete removal efficiency in the reaction process. Compared to the heterogenous reaction, the homogenous metal ions containing Fe and Co ions was almost negligible and showed 14% removal efficiency of LFX.

In addition, the role of different Co ratios in Co-BFO on LFX degradation was investigated and showed in Figure 6b. Among them, Co-BFO-2 sample showed the best photocatalytic activity in 60 min, revealing the removal efficiency increased with the Co content in the samples. This phenomenon could be attributed to the increased reactive sites by the introduction of Co element in BFO. However, further increasing of Co amount was leading to the decreased removal efficiency. The exposing surface area of Co-BFO decreased with the further addition of Co element, leading to the reduction of formation rate of photoinduced charge carriers which affected the photocatalytic activity. On the other hand, the sample with higher amount of Co element would facilitate the formation of agglomeration phenomenon, resulting in the decrease of active sites. In this case, Co-BFO-2 sample was chosen as the photocatalyst in the subsequent experiments.

### 3.3. Effect of Experimental Factors 

**Figure 7:**
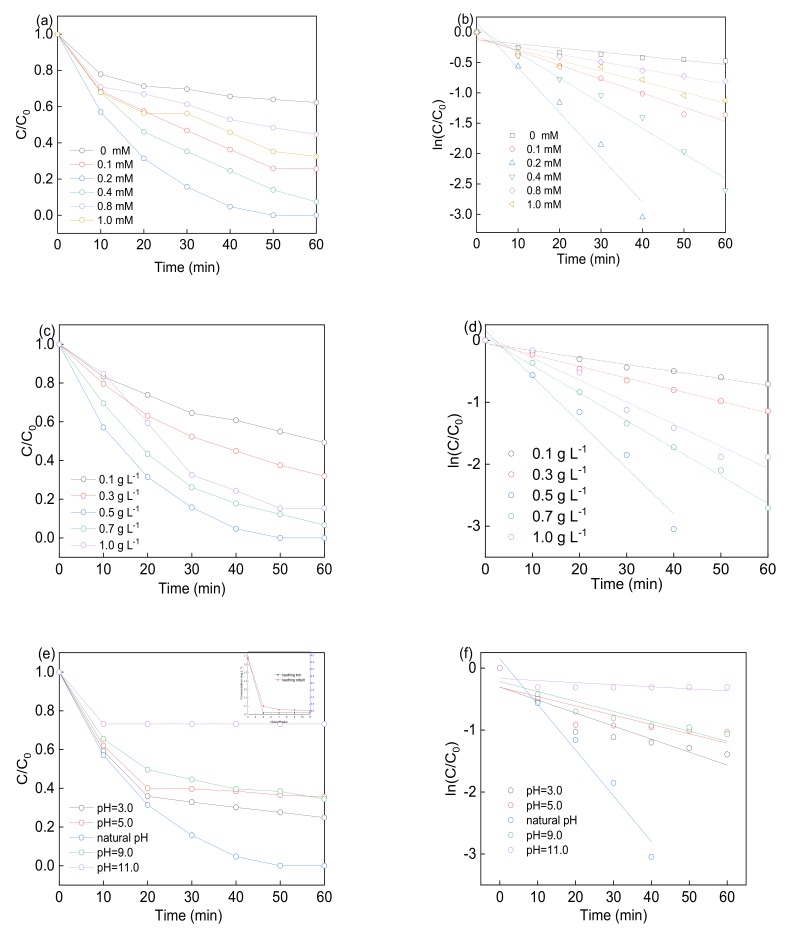
LFX degradation with different PS concentration (**a**), Co-BFO-2 dosage (**c**), initial pH value (**e**) (insert: leaching iron and cobalt concentration varies initial pH value); LFX degradation rate with different PS concentration (**b**), Co-BFO-2 dosage (**d**), initial pH value (**f**).

Figure 7a–d showed the LFX removal with different PS concentration and Co-BFO-2 dosage. The experiments were performed at 0, 0.1, 0.2, 0.4, 0.8 and 1.0 mM of PS concentration. The k_obs_ obviously increased form 6.78 × 10^−3^ to 73.8 × 10^−3^ min^−1^ with the PS concentration ranged from 0 to 0.2 mM, as well as the removal efficiency increased from 37.7% to 100%. However, the rate constant no longer increased and slightly decreased as the PS concentration further increased to 1 mM, with the LFX removal efficiency remained unchanged. It has been proved that the persulfate play dominate role in the generation of free radicals in the heterogenous PS activation process, which explained the increased removal efficiency. While the PS concentration exceed the optimal dosage, part of PS would participate in the formation of free radicals, excess PS in the reaction solution would go through the compete reaction for the free radicals according to Equations (3)–(7). Thus, the presence of overdosed PS will retard the contact chance between target pollutants and free radicals, leading to the reduction of LFX degradation efficiency [34].

Co-BFO (II) + S_2_O_8_^2−^ → SO_4_^2−^ + •SO_4_^−^ + Co-BFO(III), (3)

Co-BFO(III) + e^-^ → Co-BFO (II), (4)

•SO_4_^−^ + H_2_O → •OH + H^+^, (5)

•SO_4_^−^ + •SO_4_^−^ → S_2_O_8_^2−^, (6)

•OH + •OH → H_2_O_2_, (7)

In order to evaluate the heterogeneous catalytic activity of Co-BFO, LFX degradation was operated at natural pH in the presence of 0.2 mM PS. As can be seen in Figure 7c, the LFX degradation efficiency increased from 50.8% to 100%, and kinetic rate constant increased rapidly from 13.3 × 10^−3^ to 73.8 × 10^−3^ min^−1^ while catalyst dosage increased from 0 to 0.5 g L^−1^. However, the rate slowed and the removal efficiency was retarded from 100% to 84.8% when the catalyst dosage increased from 0.5 to 1.0 g L^−1^. With the addition of catalyst, the reaction process was significantly improved as the rise in amount of catalyst would provide more surface area and available active sites for the generation of free radicals. On the other hand, the excess catalyst dosage would limit the penetration of visible LED light and inhibit the diffusion between the target pollutant and PS catalyst. Thus, the following experiments were performed with 0.5 g L^−1^ catalyst and 0.2 mM PS on the LFX degradation.

As the reaction was performed in the aqueous solution, initial pH value was an important parameter for the LFX degradation by Co-BFO. Figure 7e showed the LFX removal efficiency by initial pH values of 3.0, 5.0, natural pH (6.6–6.8), 9.0 and 11.0. It was found that the reaction process performed effectively over a broad pH range from 3.0 to 9.0. However, it was observed that the removal efficiency was slightly improved as k_obs_ increased from 15.01 × 10^−3^ to 73.8 × 10^−3^ min^−1^ when the initial pH values changed from 5.0 to natural pH. Nevertheless, the LFX degradation efficiency reached to 75.2% and rate was 21.01 × 10^−3^ min^−1^ as the pH set at 3.0. This phenomenon could be attributed to the homogeneous iron and cobalt leaching in the acidic conditions. In this case, ICP/MS was used to detect the metal ions leaching concentration under different pH values. The leaching problem was severe compared to the other pH conditions and formed the homogenous reaction condition which responsible for the higher LFX degradation under acidic condition. On the other hand, the concentration of iron and cobalt leaching in the pH range of 6.8–11.0 were below 0.2 mg L^−1^ and showed negligible LFX degradation efficiency, which displayed the stability of Co-BFO-2 under natural and basic conditions.

### 3.4. Identification of Reactive Species and Recycling Tests 

**Figure 8:**
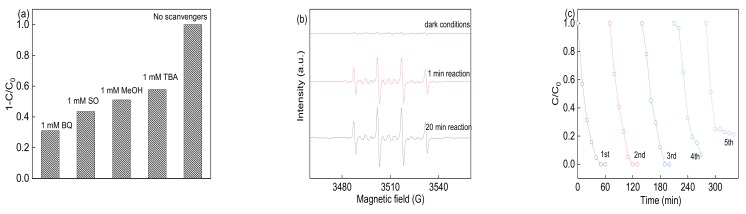
(**a**) Reactive species trapping over the Co-BFO-2 catalyst activation PS under LED light irradiation; (**b**) DMPO spin trapping ESR spectra; (**c**) recycling test for Co-BFO-2 catalyst in five times.

It has been reported that both sulfate radicals and hydroxyl radicals were of great importance in the PS activated systems, while the photogenerated holes and superoxide dominate in the photocatalysis process. In order to investigate the role of modified Co elements on the BFO in the reaction process, the radials trapping experiments were conducted by adding the individual scavengers and EPR tests. The methanol (MeOH), t-Butyl alcohol (TBA), Benzoquinone (BQ) and sodium oxalate (SO) were introduced to quench the sulfate radicals, hydroxyl radicals, superoxide radicals and photogenerated holes, respectively [35]. As demonstrated in Figure 8a, the addition of BQ and SO was of significantly effluence the reaction by Co-BFO catalyst. While the MeOH and TBA also affect the reaction process, the removal efficiency was 51.04% and 57.79%, respectively. It was assumed that the superoxide, photogenerated holes and sulfate radicals played an important role in the reaction. The sulfate radicals, holes and superoxide radicals could enhance the photocatalytic reaction. The Co elements doping in the host BFO would cause the defect in the structure, leading to generation of oxygen vacancy. 

Moreover, DMPO-trapped EPR tests were also performed to support the above speculation. As shown in Figure 8b, weak signals were observed in the presence of PS alone, revealing no free radicals occurred without the activator. However, obvious peaks of DMPO-X were obtained when the catalyst and LED light were both present in the solution, with the intensity ratio of about 1:2:2:1. The signal remained sharp after 20 min reaction, while no EPR signals were observed under dark condition. However, no obvious sulfate radical signal was detected due to the rapid transmission of sulfate radicals to hydroxyl radicals and the weak intensity compared to hydroxyl radicals. Thus, the Co-BFO-2 sample were able to produce large quantities of reactive free radicals for the PS activation under visible light irradiation.

It is essential for the heterogenous catalyst to behave good reusability and stability that be used in actual applications. The photocatalytic activity of Co-BFO was used in five successive recycles, which showed no significant decreased efficiency, which was shown in Figure 8c. The results indicated good reusability of the Co-BFO-2 catalyst during the reaction process. Meanwhile, the stability of catalyst by measurement of metal leaching was also conducted as shown in Figure 7e. In general, the cobalt and iron leaching were not significant under natural pH value. During the reaction process, the maximum cobalt leaching was 0.68mg L^−1^ and <0.2 mg L^−1^ for iron ions. It can be concluded that the Co-BFO-2 exhibited excellent reusability and stability with low metal leaching problems. Moreover, the mineralization of LFX during the process was also performed by TOC value. After 30 min and 60 min reaction, the TOC removal efficiency was about 16.77% and 36.7%, while showed more than 70% and complete removal of LFX concentration, respectively.

### 3.5. Potential Degradation Mechanism of LFX by Co-BFO 

**Figure 9:**
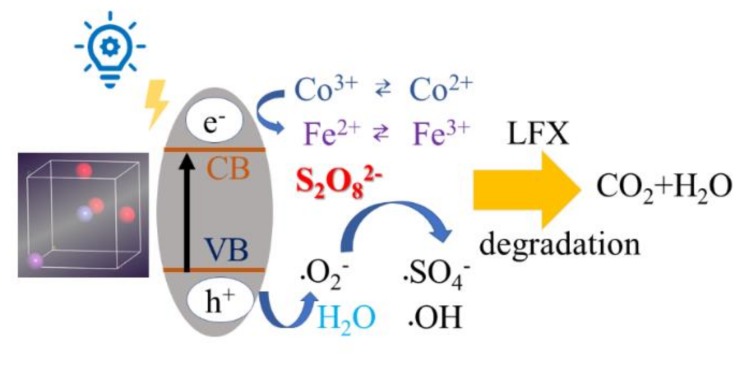
Proposed mechanism for the Co-BFO-2 catalyst in PS activation under visible LED light irradiation.

On the basis of above results, a plausible mechanism for the degradation of LFX by Co-BFO for PS activation under visible light irradiation was proposed and shown in Figure 9. According to the results, it is believed that the doping of Co into the host lattice of BFO sample by replacing Fe atoms could extend the visible light response and showed no significant structure change. By the partially replacement of Co species in the host structure, it was reasonable to expect higher efficient degradation efficiency by the utilization of Co-BFO-2. It was reported that pure BFO possessed a space group of R3c, while the refined data indicates that 1.34% of Fe was substituted by Co, which is close to the doping ratio obtained by ICP. The replacement of Fe atoms by Co ions could cause narrow bandgap by lifting the location of VB and maintaining the location of CB, as verified by UV-vis spectra, while the band gap of Co-BFO-2 was 1.50 eV, much smaller than that of undoped ones. Moreover, the Co element doped into BFO would bring structure defect in the host lattice, which further increase the oxygen vacancy on the surface, facilitating the photocatalytic process.

On the other hand, when the Co-BFO was incited by the visible light, the photogenerated electrons would be transferred to the CB band, leading the generation of holes to the VB band, which was not easily recombined. Thus, the reaction between aborted oxygen molecule and holes occurred, leading to the formation of superoxide species. Furthermore, the pairs of Co(II)/Co(III) and Fe(II)/Fe(III) would participated in the heterogenous PS activation, resulting in the formation sulfate radicals and hydroxyl radicals. Meanwhile, the presence of sulfate radicals, hydroxyl radicals and photogenerated holes and superoxide species in aqueous solution should be held accountable for the accelerated photocatalytic degradation of LFX in the heterogenous PS process under visible light.

Co-BFO (III) + LED light → h+ + e^−^, (8)

Co-BFO (III)+ e^−^ → Co-BFO (II), (9)

O_2_ + e^−^ → •O_2_^−^, (10)

•O_2_^−^+ H_2_O → •H_2_O_2_, (11)

S_2_O_8_^2−^ + •O_2_^−^ → SO_4_^2-^ + •SO_4_^−^ + O_2_, (12)

## 4. Conclusions

The magnetic and visible light active photocatalyst of BFO and Co-BFO catalyst have been successfully prepared in this experiment. As expected, the prepared Co-BFO materials showed excellent photocatalytic performance for target pollutant LFX, and the catalyst was magnetic, making it feasible to achieve separation from the solution. Among all the synthesized catalysts, Co-BFO-2 has the strongest photocatalytic performance, and the degradation rate of LFX was 3.52 times higher than that of pure BFO. The mechanism of enhancing photocatalytic performance could be ascribed to the cobalt element doping strategy, leading to the broaden adsorption edge and reduction of recombination of photogenerated electron-hole pairs by the substitution of Fe atoms. The coupled process was suitable for wide pH conditions in the solution (3.0–9.0). The synthesized Co-BFO-2 showed the best degradation efficiency, almost 100% removal and 36.7% mineralization efficiency of LFX. During the reaction process, the main active species were superoxide, photogenerated holes and sulfate radicals. The Co-BFO catalyst also exhibited well reusability and high stability during the reaction process, with extremely low Co and iron leaching concentration (<0.2 mg L^-1^, natural pH). This study provides a facile strategy to preparation of magnetic bismuth ferrite catalyst and a feasible way to improve the heterogenous PS activation under visible LED light irradiation.

## Figures and Tables

**Table 1:** Surface area, pore volume, pore size and magnetic properties of different sample.

Samples	BET Surface Area (m^2^ g^−1^)	Pore Volume (cm^3^ g^−1^)	Pore Size (nm)	Ms (emμ g^−1^)
BFO	11.8004	0.034523	3.80215	9.76
Co-BFO-1	29.9661	0.047210	3.90179	21.26
Co-BFO-2	58.1761	0.061675	4.24059	22.17
Co-BFO-3	78.8946	0.078730	3.99163	23.06

## Supplementary Materials

The following are available online at https://www.mdpi.com/1996-1944/12/23/3952/s1, Figure S1: Rietveld analysis results of BFO and Co doped BFO samples, Table S1: The structural refinement and band gap value of BFO and Co-BFO sample, Table S2 Abbreviated symbols of samples with different amount of Co, Table S3 Studies on of various contaminant using bismuth ferrite and metal doped bismuth ferrite catalysts.
