# Supplementary material for: Enhanced Activation of Persulfate by Co-Doped Bismuth Ferrite Nanocomposites for Degradation of Levofloxacin Under Visible Light Irradiation
Source: Materials (Basel). 2019 Nov 28;12(23):3952. doi: 10.3390/ma12233952 (PMC6927001; doi:10.3390/ma12233952)
Supplement: Supplementary file 1 [file materials-12-03952-s001.pdf]

materials

MDPI

Supplementary materials

Enhanced activation of persulfate by Co-doped bismuth ferrite nanocomposites for degradation of levofloxacin under visible light irradiation

Xin Zhong*, Zheng-Shuo Zou, Hu-Lin Wang, Wei Huang and Bin-Xue Zhou

Department of Environmental Science and Engineering, Beijing Normal University, Zhuhai, China; zzsslq22@163.com (Z.S. Zou); wanghulin.hu@163.com (H.L. Wang); hw_david@21cn.com (W. Huang); 26418246@qq.com (B.X. Zhou)

* Correspondence: zhongxin@whu.edu.cn; Tel.: +86-756-6126103 (X. Zhong)

Table S1. the structural refinement of BFO and Co-BFO sample.

Samples	a	b	c	Unit volume	Crystalline size	refined data
BFO	8.0278	8.4161	6.0219	406.86	41.2	1
Co-BFO-1	8.0264	8.4124	5.985	404.11	42.1	_
Co-BFO-2	8.0263	8.418	5.983	404.24	42.3	0.013909
Co-BFO-3	8.0261	8.417	5.978	403.85	44.6	_
Co-BFO-4	8.0154	8.412	5.94986	401.17	46.6	_

Table S2. Abbreviated symbols of samples with different amount of Co.

Sample abbreviation	Cobalt percentage	Stoichiometric molar ratio Bi:Fe:Co	Chemical Formula
BFO	0	1:2:0	$Bi_2Fe_4O_9$
Co-BFO-1	0.01	1:1.98:0.02	$Bi_2Fe_{3.96}Co_{0.04}O_9$
Co-BFO-2	0.02	1:1.95:0.05	$Bi_2Fe_{3.9}Co_{0.1}O_9$
Co-BFO-3	0.03	1:1.93:0.07	$Bi_2Fe_{3.86}Co_{0.14}O_9$
Co-BFO-4	0.05	1:1.9:0.1	$Bi_2Fe_{3.8}Co_{0.2}O_9$

Table S3. Studies on of various contaminant using bismuth ferrite and metal doped bismuth ferrite catalysts.

No.	Systems	Light source	Target pollutant	Reaction time and efficiency	Operation parameters
1	$Co/BiFeO_3$ composite/ peroxysulphate	none	tetracycline	60 min 81.09%	[peroxysulphate]=20 mg
2	$Bi_2Fe_4O_9$/peroxymonosulfate	none	sulfamethoxazole;	30 min >95%	pH =3.8, [PMS]=0.4 mM, [$Bi_2Fe_4O_9$] =0.1 g L^{-1}, and [SMX] =20

					μM
3	0.5/$Bi_{25}FeO_{40}$/$Bi_2Fe_4O_9$/visible light	500 W Xe lamp	Rhodamine B	300 min 70%	10 mg/L RhB, [catalyst]=1.0 g L^{-1}
4	$Bi_2Fe_4O_9$/ peroxymonosulfate	none	sulfamethoxazole	30 min k=0.14 min^{-1}	natural pH, [catalyst] = 0.1 g L−1, [PMS] = 0.40 mM, CSMX = 20 M
5	Ag/$Bi_2Fe_4O_9$/visible light	500 W tungsten lamp	methyl orange (MO)	45 min 100%	[catalyst]= 3.0 g L^{-1}
6	Co-doped $BiFeO_3$/visible light	300 W Xe lamp	methyl orange (MO)	120 min 89.8%	[MO]=10 mg L^{-1}, [catalyst]=1.0 g L^{-1}
7	Er-doped $BiFeO_3$/visible light	300 W Xe lamp	tetracycline hydrochloride	180 min 75.8%	[catalyst]=2.0 g L^{-1}, [TC]=30 mg L^{-1}
8	Sr and Co co-doped $BiFeO_3$/visible light	300 W Xe lamp	methyl orange (MO)	180 min 76%	[MO]= 5 mg L^{-1},
9	Co-BFO/PS/LED light **(this work)**	LED light	Levofloxacin (LFX)	60 min 100%	[LFX] = 15 mg L^{-1}, [Co-BFO] = 0.5 g L^{-1}, [PS] = 0.2 mM.

1. H. Zhang, S. Cheng, B. Li, X. Cheng, Q. Cheng, Fabrication of magnetic Co/$BiFeO_3$ composite and its advanced treatment of pharmaceutical waste water by activation of peroxysulphate. Separation and Purification Technology 202 (2018) 242-247.
2. W.D. Oh, V.W.C. Chang, T.T. Lim, A comprehensive performance evaluation of heterogeneous $Bi_2Fe_4O_9$/peroxymonosulfate system for sulfamethoxazole degradation. Environ Sci Pollut Res (2019) 26:1026-1035.
3. G. Wang, D. Cheng, T. He, Y. Hu, Q. Deng, Y. Mao, S. Wang, Enhanced visible-light responsive photocatalytic activity of $Bi_{25}FeO_{40}$/$Bi_2Fe_4O_9$ composites and mechanism investigation. Journal of Materials Science: Materials in Electronics (2019) 30:10923-10933.
4. W.D. Oh, Z. Dong, G. Ronn, T.T. Lim, Surface–active bismuth ferrite as superior peroxymonosulfate activator for aqueous sulfamethoxazole removal: Performance, mechanism and quantification of sulfate radical. Journal of Hazardous Materials 325 (2017) 71-81.
5. K. Wang, X. Xu, L. Lu, A. Li, X. Han, Y. Wu, J. Miao, Y. Jiang, Magnetically recoverable Ag/$Bi_2Fe_4O_9$ nanoparticles as a visible-light driven photocatalyst. Chemical Physics Letters 715 (2019) 129-133.
6. Z. Li, L. Cheng, S. Zhang, Z. Wang, C. Fu, Enhanced photocatalytic and magnetic recovery performance of Co-doped $BiFeO_3$ based on MOFs precursor. Journal of Solid State Chemistry 279 (2019) 120978.
7. J. Zhou, L. Jiang, D. Chen, J. Liang, L. Qin, L. Bai, X. Sun, Y. Huang, Facile synthesis of Er-doped $BiFeO_3$ nanoparticles for enhanced visible light photocatalytic degradation of tetracycline hydrochloride. Journal of Sol-Gel Science and Technology (2019) 90:535-546.
8. F.L. Wang, Y. Li, N. Wang, L. Zhu, A. Jain, Y.G. Wang, F.G. Chen, Enhanced magnetic, ferroelectric and optical properties of Sr and Co co-doped $BiFeO_3$ powders. Journal of Alloys and Compounds 810 (2019) 151941.

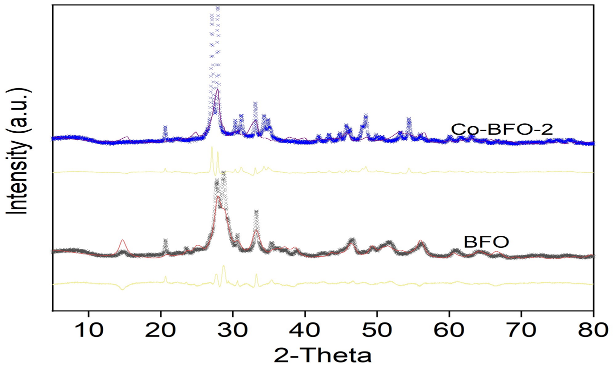

Figure S1. Rietveld analysis results of BFO and Co doped BFO samples.
